# Surgical Margins for Ameloblastoma in Dogs: A Review With an Emphasis on the Future

**DOI:** 10.3389/fvets.2022.830258

**Published:** 2022-03-22

**Authors:** Stephanie Goldschmidt

**Affiliations:** Department of Veterinary Clinical Sciences, College of Veterinary Medicine, University of Minnesota, St. Paul, MN, United States

**Keywords:** ameloblastoma, canine acanthomatous ameloblastoma, odontogenic, pathology, oral surgery, neoplasia, surgical margins

## Abstract

Ameloblastoma is a benign epithelial odontogenic tumor with the capacity to aggressively invade the surrounding bone. Surgical removal of the tumor can result in extended disease-free interval (cure). However, controversy surrounds the most appropriate surgical margin required to prevent local recurrence while simultaneously minimizing morbidity. *En bloc* excisional surgery carries the risk of major complications such as mandibular drift, hemorrhage, and oronasal fistula formation. Conservative therapy without a safety margin reduces potential morbidity but is likely to result in local recurrence. No reliable rate, nor time to recurrence, is documented but may be as high as 91% with conservative therapy. Conversely, surgery with a 10- to 20-mm margin is associated with a 0–4.6% recurrence rate. There is no documented difference in the recurrence rate with a 10- vs. 20-mm margin. The correlation of the histologic margin with the recurrence rate following excisional surgery has not determined a required histologic safety margin. Rather, no local recurrence occurs despite narrow or incomplete margins. Thus, pathologic margins > 0 mm may be sufficient to prevent local recurrence or recurrence may be protracted. Accordingly, a narrow (5–10 mm) gross surgical margin may be the most appropriate. Additional research is required for confirmation, and only level 4 evidence on safety margins has been achieved thus far. Future work should focus on defining the extension of neoplastic cells past the demarcation of ameloblastoma on variable diagnostic imaging modalities as well as determining the recurrence rate with various surgical and histologic safety margins.

## Introduction

Ameloblastoma is a benign epithelial odontogenic tumor believed to arise from epithelial cell rests that remain in the gingiva, periodontal ligament, and bone following odontogenesis. Clinical presentation and diagnostic imaging features of ameloblastoma are varied in dogs (1–3) potentially representing different biologic variations of the tumor. Ameloblastoma, specifically canine acanthomatous ameloblastoma (CAA), is common accounting for up to 45% of reported odontogenic tumors (4, 5). Despite its common nature, controversy surrounds the most appropriate surgical treatment.

The aim of surgery for ameloblastoma is to eradicate all neoplastic tissue and successfully prevent local recurrence. The metastatic risk of ameloblastoma in dogs appears to be null, with locoregional or distant metastasis not reported in most studies (4, 6–12). There is one report of pulmonary metastasis from a maxillary ameloblastoma (13), but pathologic confirmation of the pulmonary nodule was not obtained. There is also one report of pathologically confirmed thoracic metastasis with an amyloid producing odontogenic tumor (APOT) (14). Immunohistochemical evidence supports the fact that APOT may represent a variant of ameloblastoma (15). Thus, ameloblastoma in dogs may carry a small metastatic risk similar to the 2% metastatic risk in human ameloblastomas (16). Nevertheless, given the exceedingly rare propensity for metastasis, the surgical goal for ameloblastoma in dogs is preventing local recurrence while simultaneously minimizing morbidity.

Reported surgical treatments for ameloblastoma in dogs range from conservative surgery with no safety margin to excisional surgery with a 20-mm gross margin (6–13). Variable treatment paradigms are also reported in humans (17–29). Canine and human ameloblastoma exhibit a high degree of transcriptional homology (30), and surgical strategies may be similar. The aim of this article is to review the literature on the local recurrence rate associated with variable surgical and histopathologic safety margins for ameloblastoma in dogs. Due to paucity of studies with high-quality evidence, systematic review is not possible. Human literature is presented for comparison. Proposed suggestions on the appropriate safety margins are based on current evidence. Recommendations on future developments necessary to improve surgical treatment are highlighted.

### Definition of the Surgical and Histologic Safety Margins

The concept of removing a surgical margin around ameloblastoma is based on treatment algorithms for malignant tumors. Malignant cells have the propensity to extend past what is observed grossly and on diagnostic imaging. If all malignant cells are not removed, then local recurrence as well as locoregional and distant metastasis can occur. Thus, curative intent procedures remove a margin of peritumoral tissue to remove all possible aberrant cells undetected in preoperative planning (29, 31, 32). The surgical margin of excised normal tissue around the grossly appreciable neoplasm is often referred to as the safety margin.

Conversely, there is also the margin of normal tissue that is determined microscopically after excision. The surgical and histologic margins are related but are rarely, if ever, identical. There may be skip metastasis and extension of neoplastic cells separate from the main tumor that can only be identified microscopically. Furthermore, there is up to 50% contraction of soft tissue after excision and formalin fixation (33). Both factors result in a decreased histologic margin compared to the executed surgical margin.

The purpose of the surgical safety margin is to obtain a sufficient histologic margin, specifically, to obtain the histologic safety margin (HSM) defined as the margin required to prevent, or significantly decrease, local recurrence. The HSM is determined clinically by comparing the local recurrence rate to the pathologic margin (31, 32, 34, 35). Thus, it also considers the inherent flaws in pathologic review. It would be near impossible for a pathologist to review all cut margins for residual tumor cells. In fact, it has been estimated that up to 4,000 sections would be required to assess the entirety of a 10-mm tumor excised with 20-mm margins (36). The HSM addresses this limitation and identifies the tumor-free margin based on several representative slices where local recurrence is unlikely. To rephrase, it determines the histologic margin where additional therapy (revision surgery or adjuvant radiation therapy) would not be clinically recommended.

### Surgical Removal Without a Safety Margin

To the author's knowledge, only 2 studies exist that report a recurrence rate associated with conservative treatment in dogs (11, 12). Marginal removal of only the gingival portion of the lesion was associated with robust and rapid recurrence. Specifically, 91% of lesions recurred within 32 days (12). Removal of both the gingival and bony aspect of CAA via curettage was associated with 100% recurrence at 12 and 15 months following therapy (11).

Enucleation as a conservative treatment option is well documented in the human literature (17–21, 23–27). Enucleation is performed alone or followed by osteoplasty of the surrounding bone, liquid nitrogen cryotherapy, or application of Carnoy's solution (25, 26). Enucleation alone is associated with an up to 90% local recurrence rate (18, 25, 28). Recurrence is variably decreased with the addition of adjuvant treatments. Recent metanalysis reported the combined relative recurrence with enucleation +/- adjuvant therapy to be 3.15-fold greater compared to surgery with a bone margin (18). Another recent metanalysis reported pooled recurrence of 41 and 8% with conservative and excisional therapy, respectively (19).

Despite the increased risk of recurrence, some argue that conservative surgery is the appropriate first-line treatment in humans to avoid the possible morbidity of excisional surgery. They suggest that conservative treatment is followed by active surveillance with diagnostic imaging, and re-treatment is performed when, and if, required (25, 26). The risk of adopting a similar treatment algorithm in dogs is multifaceted. First, most human ameloblastomas originate within the mandibular cancellous bone and are treated prior to cortical bone perforation. This type of lesion is more amenable to complete enucleation. In fact, some published treatment algorithms state that ameloblastomas with no thinning or perforation of the cortical bone are the only lesions amenable to a conservative approach (27). Second, it is unlikely that our veterinary patients will undergo rigorous active surveillance resulting in undocumented local recurrence. When recurrence is noted, surgery will likely be more challenging and carry a higher morbidity. In humans, this is the exact argument against this “stepwise” approach. Despite ~50% of recurrences occurring in the first 5 years, recurrence has been documented up to 20+ years following surgery, and it is unclear that proper surveillance is continued past the initial postsurgical period (29).

### Surgical Treatment With a Safety Margin

Curative intent surgery requires excision with an anatomic barrier layer and a linear margin. The appropriate linear margin required to prevent local recurrence and provide a “cure” for ameloblastoma in dogs is unknown (6). Suggested surgical margins in the literature range from 10 to 20 mm (6–10). Although absent in literature, anecdotally, surgeons also report use of a smaller margin closer to 5 mm, which is often followed by osteoplasty of the surrounding normal bone. The reported recurrence rate following curative intent surgery ranges from 0 to 11% (Table 1). The highest was from a study focused solely on maxillary ameloblastomas where the gross margin was listed as “1 cm if possible” (13). Studies where a 10- to 20-mm gross margin was achieved report a 0–4.6% recurrence rate (6–11). There is no documented difference in outcome with a 10- vs. 20-mm margin. However, only one report has specifically evaluated this statistically (6). The available literature achieves level 4 evidence that surgery with a linear margin results in extended disease-free interval (cure) based on the criteria published by the Oxford Center for Evidence-Based Medicine (37).

**Table 1:** Studies that evaluated the recurrence rate of ameloblastoma following surgery with a safety margin.

**Sample size**	**Diagnostic imaging**	**Surgery performed**	**Surgical margin**	**Histologic margin**	**Follow-up**	**Recurrence rate**	**Time to recurrence**	**Distant metastasis**	**Reference**
14 dogs (15 cases)	Radiographs or CT	RIM excision	No standard surgical margin. All PDL of associated tooth removed and osteoplasty after excision. Left ventral cortex intact	Not reported	By telephone on 9/15 cases. Follow up ranged from 7 months-3.5 years postoperatively	0%	N/A	0%	(7)
25	Radiographs	Mandibulectomy	At least 10 mm beyond radiographic limit of tumor	Not reported	Reexamined by DVM at 1, 3, and 6 months postoperatively. After 6 months followed up by telephone. Median follow-up 22 months	0%	N/A	0%	(8)
42	Radiographs	Mandibulectomy	At least 10 mm beyond radiographic limit of tumor	Not reported for most. Case with recurrence was incomplete (<0 mm)	In person or by telephone. No median follow-up time listed, but goal was 12 months	2.4%	Not reported	0%	(9)
18	Radiographs	Maxillectomy	10 mm when possible	Not reported	In person or by telephone. No median follow-up time listed, but goal was 6 months	11.1%	(*n* = 2) 9 months, 18 months	5.5%	(13)
43	CT if imaging was performed	Not clarified what surgery was performed for ameloblastoma	20 mm was the definition for curative intent surgery. Unclear if this was always performed for ameloblastoma	83.7% had margin information. 91.7% complete (>0 mm). 8.3% incomplete (<0 mm). Both that recurred had complete margins	Medical record review and telephone follow-up for up to 2 years postoperatively	4.6%	(*n* = 2) 4 months, 13.5 months	0%	(10)
23	CT scan (87%) or radiographs (13%)	Mandibulectomy or maxillectomy	14/23 had gross margin listed. 42.8% 10 mm, 28.6% 15 mm, 28.6% 20 mm	34.8% complete (> 5 mm), 43.5% narrow (1–5 mm), 21.7% incomplete (<1 mm)	Medical record review and telephone follow-up. Inclusion criteria were at least 12-month follow-up. Median follow-up 33 months.	0%	N/A	0%	(6)

#### Defining the Appropriate Linear Margin

It is in the patient's best interest to utilize the narrowest linear margin sufficient to prevent recurrence while simultaneously minimizing morbidity. Although excisional surgeries are generally well tolerated, they do carry a risk, with a recent article reporting a 37.3% complication rate with maxillectomies and mandibulectomies (38). Thus, whenever possible, a more conservative approach, especially one that can maintain mandibular continuity, is desirable. Of note, CAA has a propensity for the rostral mandible (4, 6). The majority of reported complications with mandibulectomy specifically are minor and self-limiting (38). Rostral surgeries are also less likely to result in mandibular drift. Therefore, morbidity with excisional surgery may be less of a concern with ameloblastoma in this region.

There is level 4 evidence that excising normal tissue 10 mm beyond the radiographic limit of the tumor is sufficient to prevent local recurrence (6). Although suspected, it remains unclear if a more conservative margin would result in the same outcome. To define appropriate surgical margins, the capacity and accuracy of various diagnostic modalities in the determination of the true extent of a tumor are required. In humans, for example, histopathologic review of ameloblastoma after surgical intervention revealed tumor extension 2–8 mm (mean 4.5 mm) from the radiographic demarcation of the tumor (29). Based on this, most surgeons utilize a 10- to 15-mm safety margin from the radiographic limit of the tumor when the goal is curative intent surgery. Use of more advanced imaging and intraoperative techniques has also been explored to better guide surgical decision-making (39). Similar studies are required in canines. Of particular interest would be the investigation of novel imaging modalities such as positron emission tomography/computed tomography (PET/CT) and near-infrared fluorescence (NIRF). Following evaluation of tumor extension, prospective clinical studies are required to evaluate disease-free interval following surgery utilizing surgical margins guided by the aforementioned work.

#### Removing the Tooth Alveolus as Part of the Safety Margin

It is widely believed that oral tumors, especially odontogenic tumors, may track down the periodontal ligament (PDL) necessitating *en bloc* excision of the alveolus to ensure removal of all neoplastic cells (40). However, to the author's knowledge, there is no evidence to support that ameloblastomas preferentially spread in this fashion, nor any literature that supports an increased recurrence rate when a tooth is transected. Furthermore, it was initially proposed that acanthomatous epulis (now termed CAA) was of periodontal origin (41) supporting the belief that complete removal of the PDL is required to eradicate neoplastic cells. Although literature strongly supports odontogenic epithelial inclusions as the most likely origin of ameloblastoma in both humans and dogs (24, 30, 41), the exact location of these inclusions (PDL vs. gingiva vs. alveolar bone) has not been conclusively determined (41), questioning this historical dogma.

From a practical standpoint, the entire tooth alveolus where the tumor is centered will most often be excised *en bloc* if a 10-mm safety margin is utilized. In rare cases where the executed surgical margin would transect the tooth of origin, there is only level 5 evidence that leaving residual PDL increases the risk of local recurrence.

### Histologic Safety Margin

Two canine studies have pathological correlation with local recurrence. Goldschmidt et al. reported no local recurrence with incomplete (<1 mm) or narrow (<5 mm) margins (6). Sarowitz et al. also reported no local recurrence with incomplete margins (<0 mm). However, there was a 4.6% recurrence rate with “complete” pathologic margins (10). Complete margins were defined as > 0 mm making it impossible to quantify if the HSM was achieved. It is also possible there was recurrence despite “clean” margins due to field cancerization, or presence of dysplastic precancerous cells at the margin, as described for squamous cell carcinoma (42).

Level 4 evidence has been achieved that the HSM required is very narrow and/or recurrence is protracted. This author suspects margins >0 mm, i.e., no cells directly at the cut margin, prevent local recurrence for ameloblastoma in dogs. However, limitations of the current evidence must be considered while interpreting the results. The data stem from 2 retrospective case series (*n* = 66 combined), which did not have standardized postoperative surveillance and did not require diagnostic imaging for confirming disease-free interval. Furthermore, recurrence may have occurred out with the confines of the study (>12–24 months). Prospective studies, or more robust retrospective analysis, are required. Future studies should also quantify the time to local recurrence. If recurrence is significantly protracted, this may negate the recommendation for additional local therapy regardless of margin status following curative intent procedures in older patients.

### Local Recurrence With Different Variants

Ameloblastoma in humans has been historically categorized into both pathologic and biological variants. Biological variants account for pathologic pattern but are primarily focused on the imaging and clinical features of the tumor (16). Variable treatment has been traditionally recommended for different biologic variants, although this is becoming more controversial.

Pathologic patterns of ameloblastoma reported in dogs include follicular, plexiform, and desmoplastic. Within the follicular subtype, if the central cells show squamous differentiation, the tumor is classified as acanthomatous ameloblastoma (CAA). Any pathological subtype may also have a variable degree of keratinization and may be termed keratinizing ameloblastoma, although this term is not widely utilized (43).

There are two suggested biologic variants, CAA and conventional ameloblastoma, based on documented differences in clinical presentation and diagnostic imaging features (1). Specifically, conventional ameloblastomas appear to arise from an intraosseous location, are primarily located in the maxilla, and routinely have a cystic component on diagnostic imaging (1). Conversely, CAA occurs primarily in the rostral mandible and may arise from an intraosseous or extraosseous location (2). CAA does not have a predictable pattern on diagnostic imaging, especially regarding the severity of bone lysis. Primarily intraosseous lesions tend to exhibit more aggressive features on computed tomography (CT) (2, 3). Despite the suggestion, the stratification of ameloblastoma into biologic variants is not widely accepted or proven in dogs. It remains unclear if there is truly a difference in tumor biology, necessitating different treatment, or the differences in clinical presentation are secondary to other patient-specific factors.

To the author's knowledge, there is no reported difference in the local recurrence rate with different pathologic or biologic variants of ameloblastoma in dogs. It has been suggested that CAAs that arise within the cancellous bone, compared to those that arise within the gingiva, may have different biologic behavior, yet it remains unclear if these require different treatment recommendations (2, 3).

In humans, there is contradiction within the literature regarding if pathologic variants warrant different treatment recommendations (44, 45), and the relevance of pathologic variants is currently being questioned. Biological variants, conversely, have stronger evidence for varied safety margins with unicystic lesions most often treated conservatively. However, recent literature supports the fact that the treatment employed remains the most impactful influence on the recurrence rate with excisional therapy having a better outcome in unicystic lesions (19, 45). This author suspects that the same is true in dogs, and if variants do exist, surgery with a safety margin should be the standard of care regardless of classification.

## Discussion

The body of evidence supports that conservative surgery is inappropriate and a surgical safety margin is required to prevent recurrence of ameloblastoma. In fact, excisional surgery has the potential to be curative for ameloblastoma and is associated with a <5% local recurrence rate. Caution should be employed that recurrence may have been undiagnosed due to lack of standardized follow-up or may have occurred beyond the confined of timeframe of the studies. However, as most patients treated for ameloblastoma are geriatric, protracted recurrence may be of no clinical concern.

The minimum surgical margin required to prevent recurrence (obtain the HSM) is unknown. As the HSM appears to be very narrow, this author suspects that the corresponding surgical safety margin is likely between 5 and 10 mm when advanced diagnostic imaging is used for surgical planning. There are numerous gaps in the literature on the biologic behavior of ameloblastoma following surgery in dogs, and only low-level evidence is achieved regarding both surgical and histologic margin. Future work should focus on defining the extension of neoplastic cells past the demarcation on various imaging and intraoperative techniques. This will allow the determination of the gross margin that is likely required to eradicate all possible neoplastic cells. Prospective clinical trials can then correlate the surgical margin with the disease-free interval. In the interim to future work, a margin of 10 mm should be achieved whenever possible. When the histologic margin is narrow, or incomplete, clinical monitoring including diagnostic imaging rather than revision surgery or radiation appears appropriate.

## Author Contributions

SG: concept design and manuscript preparation.

## Conflict of Interest

The author declares that the research was conducted in the absence of any commercial or financial relationships that could be construed as a potential conflict of interest.

## Publisher's Note

All claims expressed in this article are solely those of the authors and do not necessarily represent those of their affiliated organizations, or those of the publisher, the editors and the reviewers. Any product that may be evaluated in this article, or claim that may be made by its manufacturer, is not guaranteed or endorsed by the publisher.
